# Royal Jelly Components Encapsulation in a Controlled Release System—Skin Functionality, and Biochemical Activity for Skin Applications

**DOI:** 10.3390/ph15080907

**Published:** 2022-07-22

**Authors:** Eleni Spanidi, Sophia Athanasopoulou, Angeliki Liakopoulou, Angeliki Chaidou, Sophia Hatziantoniou, Konstantinos Gardikis

**Affiliations:** 1 Research and Development Department, APIVITA SA, Industrial Park Markopoulo Mesogaias, 19003 Athens, Greece; spanidi-e@apivita.com (E.S.); athanasopoulou-s@apivita.com (S.A.); 2 Laboratory of Pharmaceutical Technology, Department of Pharmacy, School of Health Sciences, University of Patras, 26504 Patras, Greece; aliakop@upatras.gr (A.L.); angelina.chaidou@gmail.com (A.C.); sohatzi@upatras.gr (S.H.)

**Keywords:** royal jelly, controlled release, liposome, 10-hydroxy-2-decenoic acid (10-HDA), polyphenols

## Abstract

Royal jelly is a yellowish-white substance with a gel texture that is secreted from the hypopharyngeal and mandibular glands of young worker bees. It consists mainly of water (50–56%), proteins (18%), carbohydrates (15%), lipids (3–6%), minerals (1.5%), and vitamins, and has many beneficial properties such as antimicrobial, anti-inflammatory, anticancer, antioxidant, antidiabetic, immunomodulatory, and anti-aging. Royal jelly has been used since ancient times in traditional medicine, cosmetics and as a functional food due to its high nutritional value. The main bioactive substances are royalactin, and 10-hydroxy-2-decenoic acid (10-HDA). Other important bioactive molecules with antioxidant and photoprotective skin activity are polyphenols. However, they present difficulties in extraction and in use as they are unstable physicochemically, and a higher temperature causes color change and component degradation. In the present study, a new encapsulation and delivery system consisting of liposomes and cyclodextrins incorporating royal jelly has been developed. The new delivery system aims to the elimination of the stability disadvantages of royal jelly’s sensitive component 10-HDA, but also to the controlled release of its ingredients and, more particularly, 10-HDA, for an enhanced bioactivity in cosmeceutical applications.

## 1. Introduction

Recent years have seen growing interest in natural products to promote health, wellbeing, and beauty as these products have been shown to reduce the risk of chronic diseases and are preferred over drugs [1]. All the bee products, such as honey, propolis, bee pollen, beeswax, bee venom, and royal jelly, have been used widely in medicine, cosmetics, dietetics, and folk-traditional medicine [1,2,3]. 

One of the most attractive and popular functional foods is royal jelly, used not only in dietetics but in cosmetics too [2]. Royal jelly is a yellowish white creamy glandular secretion that is produced by the hypopharyngeal glands of nurse bees, young worker bees from the species *Apis mellifera* L. [1,4,5]. From hatching to the third day, all the bee larvae are fed exclusively on royal jelly. The larvae that are going to become queens and the queen bees are fed exclusively with royal jelly throughout their lifetime. Royal jelly has an important impact on the size and the life span of a queen bee. The queen bee is double the size of the worker honeybee, and can live up to five years, compared with the worker honeybee which lives around 45 days. Furthermore, the queen bee has developed functional sexual organs and is able to spawn more than 2000 eggs per day [6,7]. The royal jelly is a viscous, slightly acidic matter (pH 3.5–4.5), with a slightly sweet taste and special aroma [7]. Royal jelly mainly consists of 50–56% water, and of important compounds such as proteins (18%), carbohydrates (15%), lipids (3–6%), minerals (1.5%), vitamins, and free amino acids [7,8,9]. Royal jelly contains minerals such as iron, calcium, sodium, zinc, copper, manganese, and potassium.

Folic acid, riboflavin, biotin, thiamine, niacin, and pyridoxine are the main vitamins in royal jelly, and vitamins C, D, A, and E are present in smaller amounts [7,10].

Major royal jelly proteins (MRJP) together with the unsaturated fatty acid 10-hydroxy-trans-2-decenoic acid (10-HDA), found in nature only in royal jelly, are its main bioactive compounds and markers to validate its quality. Some studies have highlighted certain amino acids, amines, carbohydrates, and vitamins as potential markers [11]. Among all of them, 10-HDA is the main marker for RJ quality and authenticity [12].

These unique royal jelly components own various pharmacological effects [2,7,13,14]. For instance royal jelly possesses anti-inflammatory action attributed in a molecular level to the inhibition of the expression of TNF-α and pro-inflammatory proteins such as COX-2 and cytokines IL-1β and IL-6 [15]. Compounds of royal jelly such as jelleins, royalisin, royalactin, and apisimin could be classified as antimicrobial peptides (AMPs). These peptides are considered as a natural alternative to conventional antibiotics, as their interaction with negatively charged bacterial membranes leads to bacterial cell death [6]. Moreover, there are other compounds of royal jelly with antimicrobial activity out of the AMPs, such as 10-HDA, which exhibits growth-inhibitory activity against different bacteria [16]. Multiple studies have also assessed the immunomodulatory activity of royal jelly components in white cell populations. This activity is associated with the upregulation of interferon-gamma (IFN-γ), tumor necrosis factor (TNF-α), and the cytokines IL-12 and IL-18, but also with the reduction in the dendritic cell association of allogeneic T-cell proliferation and the downregulation of Interferon-α (IFN-α), cytokines such as IL-4, IL-2, IL-10 and nitric oxide (NO), related to allergic reactions [8].

The antiaging/anti-senescence activity of royal jelly components has been assessed in multiple cellular models, such as human dermal microvascular endothelial cells [17], human peripheral blood monocytes (PBMCs) isolated from young and aged human donors [18], or human embryonic lung fibroblasts (HFL-I) [19]. Importantly, various model organisms such as *Drosophila melanogaster* flies, *Gryllus bimaculatus* crickets, silkworms, *Caenorhabditis elegans* nematodes, and mice have been also utilized for the elucidation of the mechanisms through which royal jelly and its components show antiaging effects and promote longevity. As summarized by Kunugi and Ali, royal jelly and its protein and lipid ingredients downregulate insulin-like growth signaling (IlS) and mechanistic target of rapamycin mTOR pathway, upregulate the epidermal growth factor signaling (EGF) and the dietary restriction signaling. The regulation of these pathways result to the enhancement of key longevity-related downstream cellular processes such as DNA repair, autophagy, cellular detoxification, ribosomal function, rebuilding of cellular components, protection against telomere attrition, antioxidant activity, anti-inflammatory activity, stress resistance, and cell proliferation [19,20]. Specifically for the component 10-HDA, it could be characterized as anti-aging factor, as it inhibits metalloproteinase synthesis and enhances collagen synthesis [21,22]. Furthermore this compound also demonstrates whitening properties as it has been shown to be effective in inhibiting tyrosinase [23]. Already known and new beneficial effects of royal jelly components are continuously coming into the spotlight by accumulating scientific evidence. Therefore, a matter of major importance is the development of new techniques and methods for the optimal preservation of these precious components against its naturally occurring degradation. Royal jelly is particularly sensitive to light and temperature, as higher temperatures can cause color change and component degradation due to the Maillard reaction, enzymatic, and lipid-protein reactions. When in contact with air, it undergoes oxidation, and for this reason, its storage conditions are a major issue. The ideal storage temperature of royal jelly is −20 °C [6,9,24].

To date, various techniques are used to prevent degradation of royal jelly, such as lyophilization or encapsulation (CN1323587A patent). Other techniques include the use of dendritic structures for antimicrobial use, and the coating of nanoparticles [25]. Despite the new techniques that have been developed, the creation of a system with royal jelly for dermatological use is a particular challenge, as its sensitive components must first be protected from degradation caused by the external environment. Still, the system should be able to be incorporated into cosmetic formulations so that it can be ensured that there will be a lasting release of the royal jelly ingredients to the target cells, to achieve its activity on the skin. 

Cyclodextrins (CDs) are cyclic oligosaccharides with a non-polar interior cavity and a hydrophilic surface, the properties of which can be significantly modified upon chemical modification of their hydroxyl groups. Depending on the number of glucopyranose units they bear, they can be classified as α-CD, β-CD, and γ-CD. They are often used to form encapsulation complexes of bioactive substances to increase solubility in water, protect against molecular degradation and oxidation [26,27,28]. In a study by Szejtli [29], to increase the stability of royal jelly, cyclodextrin was used in combination with lyophilization. 

Another standard encapsulation structure is liposomes’ unilammelar or multilammelar structures consisting mainly of phospholipids. Liposomes bear a hydrophilic core and periphery, whereas the lipid bilayer constitutes the lipophilic part. Due to their structure they may encapsulate both hydro- and lipo-soluble molecules and protect them from degradation, increase solubility, change their release rate, and penetrate deep layers of the skin [30]. 

Furthermore, the combination of cyclodextrins with liposomes appears to result in promising delivery systems that demonstrate higher bioavailability [31] bioactivity [32,33], stability, ingredient retention [34] as well as better controlled release rates [35] compared with single-component systems.

In another study from our group, very interesting stability, bioactivity and controlled release properties were attributed to another bee product, propolis, due to its encapsulation into a combinatorial liposome/cyclodextrin system [3]. 

The combinations of liposomes with bioactive molecules conjugated in another carrier have been described in the literature as modulatory liposomal controlled release systems (MLCRS) [36] or advanced drug delivery nanosystems functioning as Modulatory Controlled Release nano Systems (MCRnSs) [37]. Such systems, due to the entropic and enthalpic interactions between host carriers and guest-host molecules, may demonstrate interesting properties concerning the stability, bioavailability, and release rate of the encapsulated materials.

The present study aims to eliminate the stability disadvantages of royal jelly, but also to achieve the controlled release of its ingredients and more particularly that of 10-HDA and the polyphenols that it contains. More specifically, the present invention exploits the special features of both carriers, namely: the ability of cyclodextrins to encapsulate polyphenols and their enhanced skin penetration ability as well as the ability of liposomes in encapsulating large amounts of components of various degrees of polarity, for local delivery of components and for controlled release rate. As also mentioned above, an increasing line of evidence has reported that royal jelly components, and especially 10-hydroxy-2-decenoic acid (10-HDA), defensin-1 and other characterized proteins of royal jelly promote synthesis of growth factors, migration of skin fibroblasts or keratinocytes for effective wound healing, reactivation of genetically silenced genes and increased cellular proliferation [38,39]. In this direction and given the enhanced protection of royal jelly’s bioactive compounds against oxidation and degradation in this novel delivery system, we also evaluated its efficacy in cell viability, proliferation, and metabolism, as well as in an array of genes related to increased fibroblast functionality. Our findings support that this double encapsulation of royal jelly’s bioactive compounds ensures and facilitates their proper delivery to their multiple cellular targets, introducing hence an ingredient with multiple cosmeceutical and health-care applications.

## 2. Results

### 2.1. Characterization of RJDS

In the stability test carried out, it seems that there is a degradation in the phenolics after the end of the 24 weeks at a temperature of 38 °C, showing a statistically significant difference between the initial value and all the other temperatures. 6 °C, seems to be the most stable temperature (Table 1).

Entrapped 10-HDA is the total 10-HDA measured in the final system (RIDS) and in the same system without cyclodextrin (RJL), as described in Section 2.3. Initial 10-HDA refers to the 10-HDA content of the raw royal jelly to be encapsulated in the system when dissolved in methanol. The encapsulation efficiency of royal jelly components incorporation in the system (RJDS) was for polyphenols is >95% and for 10-HDA >85%. The encapsulation efficiency of the same system without cyclodextrin (RJL) was for polyphenols is >40% and for 10-HAD >29%.

The 10-HDA stability test of RJDS demonstrated a degradation of 10-HDA after the end of the 24 weeks only at 38 °C, showing a statistically significant difference from the initial value. At all the other temperatures, no statistically significant changes were noticed. This fact suggests that under the right storage conditions, RJDS is able to protect 10-HDA from degradation.

Although the RJDS and the pure royal jelly had the same values of 10-HDA (without a statistically significant difference) at the beginning of the stability test, it seems that after 24 weeks, pure royal jelly is significantly degraded as far as 10-HDA is concerned (Table 2).

Τhe mean hydrodynamic diameter, polydispersity index and ζ-potential of the system at day 1 and after 1 month at various temperatures, as well as at 6 °C after 2 and 6 months, is depicted in Table 3. Room temperature (25 °C) and 38 °C experiments were ceased after 1 month as increase in particle size was observed.

For comparison reasons the RJL was measured at day 0 and the results were: z-average diameter 247 ± 61, PI 0.580 ± 0.112 and ζ-potential −22 ± 4.3.

### 2.2. In Vitro Controlled Release Studies

**Figure 1:**
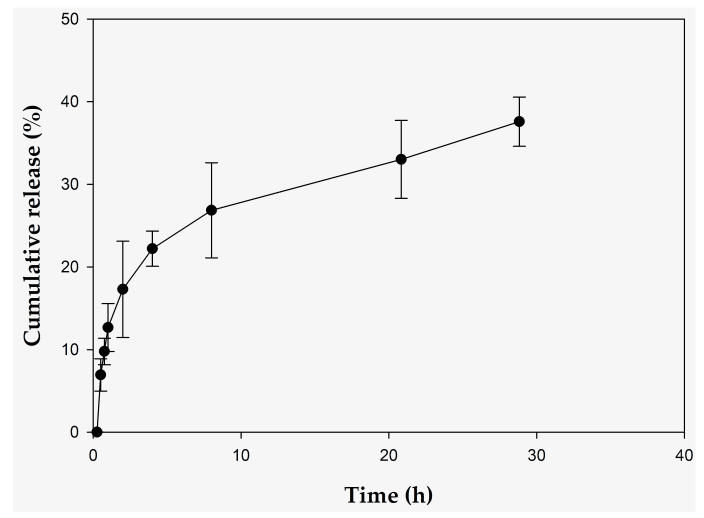
In vitro release of 10-hydroxydecenoic acid (10-HDA) of RJDS for time 0 to 8 h. The results are shown as the mean ±SD of three experiments.

The cumulative release of 10-HDA at pH 7.2 and at a temperature of 37 °C was 26.85% ± 5.76% at 8 h. In vitro release profile of RJDS as a function of time is presented in Figure 1.

The RJDS releases 22.21% ± 2.13% of ingredients (10-hydroxydecenoate acid, 10-HDA) in 4 h with 37.57% ± 2.98% release of encapsulated 10-HDA from the system in 30 h. The RJL release 82.3% ± 3.19% of 10-HDA in 4 h. 

### 2.3. In Cellulo Studies

**Figure 2:**
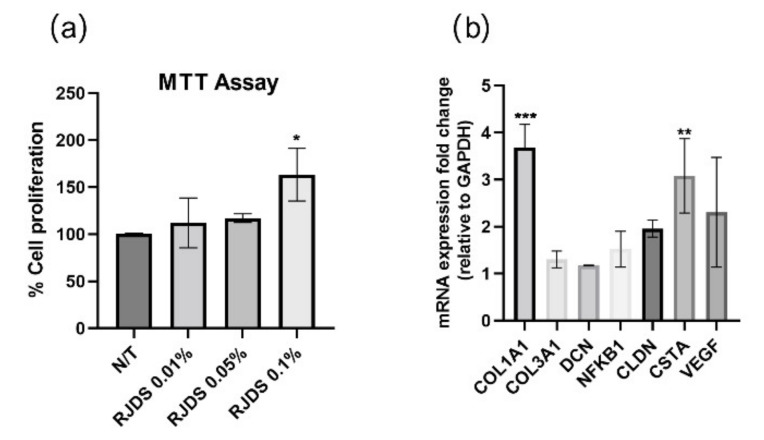
Results from in cellulo assays in NHDF cells. (**a**) Cell proliferation results based on MTT assay for untreated NHDF cells (N/T) and NHDF cells treated with different concentrations of RJDS diluted in the cell-culture medium (0.01%, 0.05%, 0.1% *v*/*v*). Value for untreated cells was arbitrarily set to 100% * *p* < 0.05 indicates groups significantly different from the control (ANOVA test—Dunett multiple comparisons test). (**b**) Relative gene expression levels for Col1A1, Col3A1, DCN, NFKB1, CLDN, CSTA and VEGF expressed as a fold change ± SEM compared to the control NHDF cells and using GADPH as internal reference gene. The experimental conditions were control (untreated NHDF cells—N/T) and cells treated with RJDS dissolved in cell culture medium in the percentage of 0.1% *v*/*v*). *** *p* < 0.001, ** *p* < 0.01 indicates groups of significantly different from the control (ANOVA). The data correspond to the mean ± SEM of three independent experiments.

The proliferative effect of the RJDS in NHDFs was examined via its administration to fibroblasts, diluted in serum-free medium in three different concentrations (0.01%, 0.05% and 0.1% *v*/*v*), compared to the control (not-treated-N/T). After 24 h of treatment, the extract demonstrated growth-promoting activity at concentration of 0.1% *v*/*v*, as shown in Figure 2a.

To gain an insight into the underlying molecular mechanisms behind the observed increase in proliferative capacity fibroblasts, we administered the RJDS in the optimal concentration for the cells, as determined by the MTT assay (diluted in complete cell culture medium in 0.1% *v*/*v*) and next we assessed transcript accumulation for a panel of selected genes involved in many skin-regeneration related processes. Gene transcripts were measured by quantitative real time RT-PCR (RT-qPCR) using respective primer sets designed from the transcribed region of each gene using Primer Express 1.5 software (Applied Biosystems, Darmstadt, Germany). Specific gene transcript levels showing statistically significant changes upon treatment with the extract were Col1a1 with a 3.68-fold change and CSTA with a 3.08-fold change, whereas Col3A1, DCN, NFKB1, CLDN, and VEGF all demonstrated a transcriptional upregulation (Figure 2b).

### 2.4. Tape Stripping

**Figure 3:**
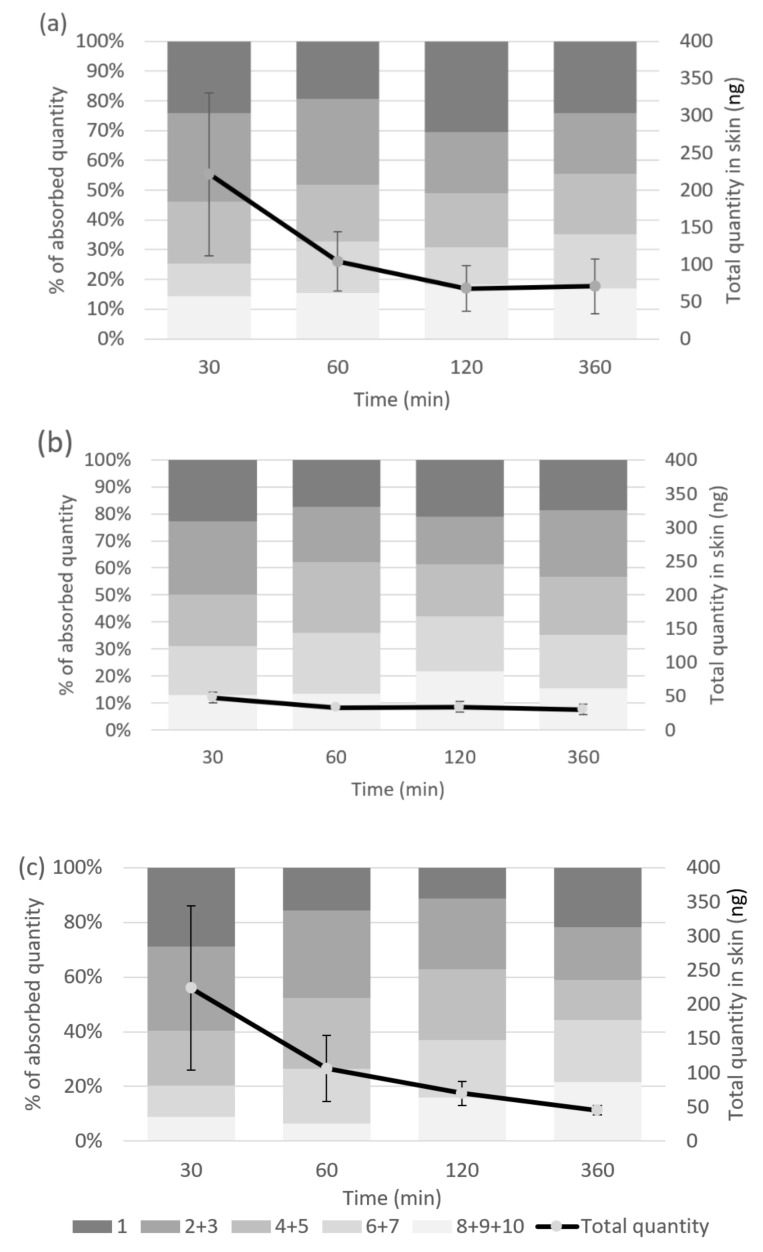
Profile of penetration of (**a**) RJDS, (**b**) RJL and (**c**) royal jelly in stratum corneum (SC) after application on the skin of healthy volunteers.

The profiles of penetration of RJDS and Royal Jelly in SC after application of the samples on the skin of healthy volunteers over time is presented in Figure 3. The total amount of 10-HDA of RJDS or Royal Jelly detected 30 min after application was 221.21 ng ± 109.69 ng and 223.80 ng ± 120.22 ng, respectively, which corresponds to 36.87 ng ± 18.28 cm^2^ and 37.30 ng ± 20.04 cm^2^ of skin area. The penetrated quantity of 10-HDA of both samples remained unaltered throughout the experiments. 30 min after application it was mainly situated on the outermost SC layers (tape 1 and tapes 2 + 3 for RJDS: 25.56% ± 11.14% and 31.93 % ± 12.29 %, respectively, and for Royal Jelly: 28.95 % ± 3.37 % and 30.72 % ± 2.84 % of absorbed quantity, respectively). The percentage of the absorbed quantity that reached the deepest examined strata of SC (tapes 8 + 9 + 10) was 13.14% ± 5.47% for RJDS with no significant change, whereas Royal Jelly reached this result at 120 min (tapes 8 + 9 + 10 at 30 min: 8.92% ± 0.66% and at 120 min 15.93 ng ± 8.20 ng). Both samples maintained the same distribution pattern at least until 360 min post application. In contrast, the penetrated quantity of 10-HDA of the RJL sample was found at a lower level than both other samples, more specifically below 50 ng in all different time points of the experiments.

## 3. Discussion

Royal jelly is a unique beehive product and one of the most attractive functional foods. It has been used since ancient times in traditional medicine and cosmetics. The main hindrance to its use is that the storage must be carried out at low temperatures and in the absence of light, and even so, the lifespan is quite short. 

In the present work, we developed a system consisting of cyclodextrins and liposomes that encapsulates and protects royal jelly’s 10-HDA for skin applications.

The measured physicochemical characteristics (size, ζ-potential) of the royal jelly system (RJDS) remain stable over time when stored at 6 °C. Moreover, the total phenolic content and 10-HDA remain also stable. In contrast with pure royal jelly from the same batch, which degrades rapidly upon all storage conditions except 6 °C, RJDS protects the easily degradable 10-HDA of royal jelly, providing a specific added value to the system. To protect 10-HDA from degradation, royal jelly should be stored in a frozen state as soon as it is harvested. It was shown that only storage at cold temperatures prevents decomposition of 10-HDA without causing no alterations in 10-HDA [12,40,41]. The same pattern was observed for RJL system with slight differences in size (smaller particles) and increased P.I. facts that can be attributed to a slight swelling of the lipid membrane due to cyclodextrin encapsulation and a possible stabilization due to lipid-carbohydrate interaction, respectively.

The main active ingredient in royal jelly (10-HDA) was determined and the release rate was 22.21% ± 2.13 at 4 h. 10-HDA was stable after 24 weeks in RJDS and showed no statistically significant differences between the different temperatures. RJDS appears to be an effective system for the protection of 10-HDA.

These assays indicate that, if maintained at the right conditions, RJDS is a stable form of encapsulating and protecting 10-HDA. The release rate of the 10-HDA that the system demonstrated is suitable for applications where a prolonged provision of royal jelly is desired, such as antiaging targeting.

To validate the bioactivity of RJDS, we administered it in mammalian cell cultures (NDHFs) by cell culture medium supplementation, in a range of different concentrations.

First, we examined the effects of different concentrations of RJDS on cellular proliferation and metabolic activity via MTT assay. Given the important observed increase in cell viability and metabolism of NHDFs treated with RJDS, we assessed the molecular activity of the extract by targeting a panel of genes representative of extracellular matrix structural proteins, in association with regulatory genes involved in pathways that modulate growth factors, cell adhesion and contractility. 

We observed an enhanced expression of Collagen1A1, one of the most important providers of structural support to the extracellular matrix and an activator of dermal fibroblast cells well-known in the art [42]. Steady expression of Collagen3A1 and collagen-remodeling proteoglycan Decorin (DCN) were also observed. This outcome, in association with the upregulation of Col1A1 is indicative of the regulatory role of RJ in normal ECM remodeling [43]. It has been recently demonstrated also in vivo that, knockout of the DCN gene leads to skin fragility and abnormal collagen morphology, characterized by uncontrolled lateral fusion of fibrils [44]. Royal Jelly has been previously proven to counteract UV-induced photoaging in normal human dermal fibroblasts, as fibroblasts treated with RJ showed increased Collagen1A1 and transforming growth factor (TGF)-β1 production in their recovery after UV irradiation, compared to untreated cells [45]. Beneficial effects of royal jelly and 10-HDA in the restoration of the extracellular matrix have been reported not only in cell cultures, but also in mammalian models, where treatment with RJ improved the ECM quality in ovariectomized rats by a reduction in collagen crosslink and stimulation of the expression of collagen-modifying enzymes [15].

In the present study, significant upregulation of CSTA expression was also demonstrated, suggesting the implication of RJDS in cell adhesion, that is of vital importance in the establishment and maintenance of multicellular structure and signal transmission [46]. The protein Claudin (CLDN1), with an important role in cell contractility and overall in the epithelial barrier structure, function, and regulation [47] also demonstrated an increasing trend. 

Interestingly, an increase in the expression of NF-Κb was also detected, as the latter has been shown to orchestrate the expression of growth factor genes, cell adhesion, angiogenesis and cell migration, in support with previous studies that have emphasized the role of royal jelly also in wound healing [48,49]. An increasing trend was also observed in the expression of VEGF, a factor that apart of being a key mediator in vascular remodeling, it has also been demonstrated that it promotes specific ECM synthesis and fibroblast activation [50]. Given this upregulation observed in the transcriptional level, proteomic experiments will further depict the effects of the extract in complex protein interactions.

The proposed underlying mechanism for the upregulation of such genes, implicated to fibroblasts’ functionality, along with the increased cell viability and proliferation is the promotion of epidermal growth factor (EGF) signaling. As assessed in bees, switching on the EGF receptor signaling pathway, ultimately leads to epigenetic changes and a long-lived queen phenotype. This epigenetic reprogramming has been mainly associated with the inhibition of histone-modifying enzymes, such as HDACs, not only in bees but in mammalian cells as well [38]. Additionally, in turn, recent evidence supports that HDACs inhibition in mammalian cells can promote survival, proliferation, and differentiation [51,52]. The same effect of increased survival was also observed in the nematode *C. elegans*, where the administration of royal jelly [53] and 10-HDA [54] prolonged its lifespan through the activation of the EGF pathway. The association of EGF activation with cell growth and proliferation has also been reported in cellular and in model organisms such as *D. melanogaster* and *C. elegans*, and those biological benefits of EGF have been utilized in medical uses for improving wound healing as well as in today’s skin cosmetics [55].

In vivo tape stripping exhibited similar overall quantity of the active that was detected in the SC for both pure royal jelly and the RJDS. No allergic reactions were recorded in the in vivo study participants. In general, for royal jelly, allergic reactions and hypersensitivity have been mainly recorded in oral consumption. It is important here to mention that the dominant allergens of royal jelly which could present a limitation to the usage of royal jelly as a therapeutic agent are MRJP1 MRJP2 and MRJP3 [56,57]. However, topical application to the skin has not been associated with allergic reactions; on the contrary, as assessed by recent studies, it has been shown to alleviate symptoms of pruritus in a murine model of allergic contact dermatitis [58], and data from clinical studies have also confirmed that it protects against epidermal stress reactions, through the upregulation of the stress-responsive gene NQO1 [59], a fact that further supports its anti-aging potential. The present royal jelly delivery system RJDS was further tested for allergens in a formula that contained RJDS as the only active ingredient in a concentration of 1% *v*/*v*, according to an EC Reg 1223/2009 and SCCS 1459 compliant analysis and a patch test that confirmed the absence of allergens.

Even though pure royal jelly and the RJDS demonstrate similar patterns of skin penetration depth and in vivo time release, the stability parameter should be taken into consideration. This means that RJDS can maintain royal jelly’s natural tendency towards penetration and release, but at the same time protecting its valuable 10-HDA from degradation.

## 4. Materials and Methods

### 4.1. Royal Jelly Encapsulation in A Liposome-Cyclodextrin Delivery System (RJDS)

To produce the RJDS, raw royal jelly from Greek cultivation was used at a concentration of 3% (*w*/*w*). Ultrapure water produced with a Reverse Osmosis System and 1.3 propanediol (Connect Chemicals, Vimercate (MB), Italy) at ratio 55/45 were used as solvents and 5% (*w*/*w*) hydroxypropyl-β-cyclodextrin (Gangwal Chemicals Pvt. Ltd., Mumbai, India) was dispersed in the solvent system. After seven days, the RJDS was filtered by 25 μm nylon bag. Under intense stirring (3000 rpm), 3% *w*/*w* liposome, Pro-LipoTM Neo (Propanediol 75.0% *w*/*w*, Lecithin 25.0% *w*/*w*, Tocopherol 0.25% *w*/*w*, Helianthus Annuus (Sunflower) Seed Oil 0.15% *w*/*w*) were added—provided by Generex Pharmacist Pvt. Ltd., Mumbai, India/Lucas Meyer Cosmetics, Massy, France. After three days at 6 °C, it was filtered through a 0.22 μm Millipore membrane filter. For comparison reasons, a similar system without cyclodextrins (RJL) was produced. The suspension was stored at 6 °C. The encapsulation efficiency in delivery systems refers to the amount of bioactive compound encapsulated in the system [60]. We used 10-HDA as a marker, calculating the encapsulation efficiency percentage as:

EE = entrapped 10-HDA/initial 10-HDA × 100(1)

#### Physicochemical Characterization of RJDS

The hydrodynamic diameter of the RJDS system was measured by light scattering as described by Gardikis et al. (2010) [61]. The liposomal suspension was 30-fold diluted immediately after preparation or after reconstitution (100 μL) and then analyzed the ζ-potential and z-average mean of the formulations were determined. RJDS system was scattered (633 nm) at 90° angle, and measurements were carried out at 25 °C in a photon correlation spectrometer (Zetasizer 3000 HS, Malvern Instruments, Malvern, UK) and analyzed by the CONTIN method (MALVERN software, version 7.01, Malvern, UK).

### 4.2. Total Phenolic Content (TPC)

The total phenolic content was determined using the Folin-Ciocalteu colorimetric method according to the method by Arnous et al. [62]. The phenols were determined using calibration curve for 100 mg/L to 1200 mg/L of Gallic Acid (GA) and absorbance was 750 nm. The samples (0.02 mL) were diluted in ultra-pure water (1.58 mL) and the followed addition of Folin-Ciocalteu reagent (0.1 mL) (Merck KGaA, Darmstadt, Germany) and mixed. After 1 min, aqueous Na_2_CO_3_ solution (20% *w*/*v*) (0.3 mL) was added and mixed by vortex. The mixture was kept for 2 h in darkness and at room temperature for 2 h, and absorbance measurement followed.

### 4.3. 10-Hydroxydecenoic Acid (10-HDA)

Quantitative analysis of 10-hydroxydecenoic acid (10-HDA) in RJDS was carried out via High Performance Liquid Chromatography (HPLC) using HPLC AGILENT HEWLETT PACKARD SERIES 1100 (Agilent Technology, Urdorf, Switzerland), according to the method by Caparica-Santos and Marcucci [63]. The system was equipped with a DAD detector and a column C18 (Kromasil 100 5 μm 4.0 × 250 mm (MO5CLB25). The mobile phase consists of 50% methanol (PanReac, AppliChem, UHPLC Supergradient ACS) and 50% ultrapure water (produced in APIVITA) with pH 2.5 adjusted with phosphoric acid (Carlo Erba, 99%) with isocratic elution at 1.0 mL/min. The column was set at 40 °C and the detector absorbed at 215 nm. The injection volume was 20 μL and the quantification of 10-HDA was accomplished by means of a calibration curve from 2.5 to 3000 ppm (R^2^ = 0.9982). The data processing was performed with a software ChemStation for LC (Agilent Technologies) version B.04.03. The background noise was checked for the RJDS and RJL with the solvent system (ultra-pure water, 1.3 propanediol and hydroxypropyl-β-cyclodextrin) without royal jelly and for tape stripping without treatment.

The RJDS and RJL were analyzed without any treatment. Pure royal jelly was dissolved in the mobile phase (methanol and water, 50:50, *v*/*v*) at a rate 3%.

### 4.4. Stability of RJDS

RJDS was stored for six months at different temperatures (25 °C, 6 °C, 38 °C and UV light) for physical and chemical stability tests. The parameters that were tested for chemical stability were total phenolic compounds by the Folin-Ciocalteu method and 10-HDA by HPLC.

### 4.5. In Vitro Controlled Release Study

In vitro release study of the 10-hydroxydecenoic acid (10-HDA) of RJDS was measured in a buffer solution pH 7.2 (CARLO ERBA Reagents, Val-de-Reuil, France) at 37 °C, by using dialysis sacks MWCO = 1000 (Pur-A-Lyzer, Midi Dialysis Kit, Sigma-Aldrich, St. Louis, MO, USA). 10-hydroxydecenoic acid (10-HDA) were examined at different time points (from 15 min to 8 h) [3].

### 4.6. Human Skin Cell Culture

Primary Normal Human Dermal Fibroblasts (NHDF) isolated from normal human adult skin were purchased from Lonza CloneticsTM (Lonza, Walkersville, MD, USA) [64]. Cells were cultured according to Lonza instructions. The cells were grown in a recommended complete medium DMEM, high glucose (Thermo Fisher Scientific, Waltham, MA, USA) that contained 10% serum and 1% penicillin/streptomycin (Thermo Fisher Scientific, Waltham, MA, USA). Cells were sub-cultured and seeded for downstream experiments once they had reached 90% confluence. Cells were treated with the tested compound (RJDS) diluted in the cell culture medium in the concentration of 0.1% *v*/*v* and cells that received only cell culture medium were used as their control.

### 4.7. MTT Cell Viability Assay

Cell viability was determined using an MTT colorimetric assay kit (Vybrant ^®^ MTT Cell Proliferation Assay Kit, Thermo Fisher Scientific, Waltham, MA, USA) following the manufacturer’s protocol. NHDF cells (100 μL) suspended in DMEM at a density of 10^4^ cells/mL were seeded onto each well of 96-well plates and incubated at 37 °C for 24 h in a CO_2_ incubator (Galaxy 48 R, New Brunswick an Eppendorf Company). The medium was replaced with serum-free medium for cell starvation for 12 h, and the cells were subsequently treated with RJDS diluted in serum free medium or serum-free medium (control) for 24 h. After cell treatment, a mix of 100 µL DMEM and 10 µL MTT labeling reagent (5 mg/mL) was added in each well, and the plate was incubated for 4 h at 37 °C. The generated formazan crystals were dissolved in 70 μL of DMSO following removal of the supernatant. Cell viability was determined by the absorbance at 570 nm using microplate reader (infinite 200M Pro, Tecan, Switzerland). Measurements were performed and the percentage of cell viability was determined as follows: Cell viability (%) = Mean OD/Control OD × 100%. Three independent experiments were performed.

### 4.8. Gene Expression Analysis

For the isolation of total RNA (500 ng) as well as for cDNA synthesis, the Nucleospin RNA kit (Macherey-Nagel, Düren, Germany) and the PrimeScript-RT reagent kit (Takara Bio, Otsu, Japan), were used, respectively. The qPCR method as well as the process of gene analysis were conducted as described before [65]. For the relative gene expression, the comparative threshold cycle (Ct) method was used [66]. The housekeeping gene Glyceraldehyde-3-phosphate dehydrogenase (GAPDH) was selected as normalization control. Two conditions are presented: untreated NHDF cells (N/T), NHDF cells treated with RJDS. Three independent experiments were performed.

### 4.9. Tape Stripping

The penetration depth of RJDS (as well as pure royal jelly and RJL—for comparison reasons) in stratum corneum (SC) was evaluated in vivo, by tape stripping technique [67]. The adessive tapes was a polypropylene film with solvent-free adhesive (tesa film 57341-00008 transparent, tesa SE, Norderstedt, Germany) cut in strips of 3 cm^2^ area. Royal jelly was used as control. A dose of 2 mg/cm^2^ of RJDS or royal jelly was applied on the skin of forearm of 6 healthy volunteers in four neighboring sites that were corresponded to a time interval (30, 60, 120 and 360 min). At the end of each time interval, the skin site was cleaned by gentle tapping with a tissue paper. A tape was adhered applying mild pressure by a roller and was removed. The procedure was repeated obtaining 10 consecutive stripping per site of application which corresponds to about 20% of total SC [67]. Two tapes were obtained from an untreated neighboring skin area and served as control. The tapes were grouped (1 alone, 2 + 3, 4 + 5, 6 + 7, 8 + 9 + 10) and added in screw cap glass vials with 4 mL ethanol and left overnight. The vials were sonicated at a bath sonicator (30 κHz) for 20 min and centrifuged at 12,000 rpm for 6 min (×2 times). Aliquots of 2 mL were collected from each vial, avoiding the adhesive material. The sample content was analyzed by HPLC-DAD, as described earlier.

### 4.10. Statistical Analysis

Results are expressed as the mean ± SD of three different experiments. Statistical analysis between two individual groups was performed by Student’s *t*-test and one-way ANOVA (Dunnett’s multiple comparisons test) A *p* ≤ 0.05 was considered statistically significant. Statistical analysis and graphs were performed with Sigma Plot Software v.10 and GraphPad Prism 9 (GraphPad software Inc., San Diego, CA, USA).

## 5. Conclusions

The encapsulation of royal jelly provides protection for its fragile ingredients. The royal jelly liposome/cyclodextrin (RJDS) suspension eliminates the stability disadvantages of royal jelly and remains stable for 6 months (24 weeks), in terms of physicochemical characteristics, polyphenols and 10-HDA retention. Furthermore, the double encapsulation of the royal jelly in cyclodextrin/liposomes allows for 10-HDA time-controlled release which could be proven useful for skin applications, as indicated by the in cellulo experiments. The latter provide first mechanistic evidence that Royal Jelly bioactive compounds encapsulated in this novel delivery system modulate ECM homeostasis by regulating both collagen production and deposition via multiple regulatory pathways, and thus contribute to increased functionality of human fibroblasts. This fact, in combination with the deep epidermis penetration and the slow in vitro release, makes this combinatorial delivery system a possible solution to the royal jelly stability issues that hinder its efficacy and applications.

## 6. Patents

Patent WO2021240183A1 “Preparation method of a colloidal system of stabilization and controlled release of royal jelly components for various uses” has resulted from data presented in this paper.

## Figures and Tables

**Table 1:** Stability test TPC: Total Phenolic Compounds (±SD), of RJDS and RJL.

Time	Parameters	RJDS TPC (μg GA/mL) ± SD			RJL TPC (μg GA/mL) ± SD		
*t* (0)		354 ^a^	±	15	150	±	15
24th week	25 °C	306 ^c^	±	16	135	±	126
	6 °C	308 ^b^	±	8	147	±	11
	38 °C	193 ^a–d^	±	34	29	±	101
	UV	282 ^d^	±	17	100.5	±	62

The values with the same letter as superscript (a–d) shows statistically significant difference, *p* ≤ 0.001.

**Table 2:** Stability test for 10-HDA: 10-hydroxy-2-decenoic acid (±SD), of RJDS versus pure royal jelly.

Time	Parameters	RJDS (ppm) ± SD			Pure Royal Jelly (ppm) ± SD			RJL (ppm) ± SD		
*t* (0)		1142 ^a, b, e, f^	±	107	1198 ^a^	±	49	317	±	14
24th week	25°C	96 ^e–l^	±	115	615 ^d, p^	±	127	N.D.		
	6°C	962 ^c, f–i^	±	148	893 ^c, j, m, p, o^	±	18	N.D.		
	38°C	816 ^g, k, n, o^	±	102	550 ^p, q^	±	35	N.D.		
	UV	949 ^b, h, l, m, n^	±	64	447 ^d, q^	±	16	N.D.		

The values with the same letter as superscript (a–q), shows not statistically significant difference, *p* ≤ 0.001.

**Table 3:** Hydrodynamic diameter, polydispersity index and ζ-potential of the system at day 1 and after 1 month at various temperatures.

System	Z-Average Diameter (nm) ± SD			PI ± SD			ζ-Pot (mV) ± SD		
RJDS day 0	301 ^a^	±	33	0.399 ^d^	±	0.044	−17	±	4.0
RJDS 6 °C week 4	308 ^b^	±	45	0.251 ^a^	±	0.065	−19	±	5.2
RJDS 25 °C week 4	449	±	101	0.404 ^e^	±	0.063	−20	±	3.9
RJDS 38 °C week 4	605 ^a, b^	±	88	0.710 ^a–e^	±	0.105	−18	±	3.8
RJDS 6 °C week 8	428	±	95	0.341 ^c^	±	0.115	−19	±	4.2
RJDS 6 °C week 24	508	±	99	0.299 s ^b^	±	0.112	−25	±	5.9

Values with the same letter as superscript (a–e) shows statistically significant difference, *p* ≤ 0.05.

## Data Availability

Data are contained within the article.

## References

[1] Guo J., Wang Z., Chen Y., Cao J., Tian W., Ma B., Dong Y. (2021). Active components and biological functions of royal jelly. J. Funct. Foods.

[2] Ramadan M.F., Al-Ghamdi A. (2012). Bioactive compounds and health-promoting properties of royal jelly: A review. J. Funct. Foods.

[3] Spanidi E., Karapetsas A., Voulgaridou G.P., Letsiou S., Aligiannis N., Tsochantaridis I., Kynigopoulos S., Lambropoulou M., Mourtzinos I., Pappa A. (2021). A new controlled release system for propolis polyphenols and its biochemical activity for skin applications. Plants.

[4] Anil S., Dosler S., Mericli A.H. (2016). Chemical composition and antimicrobial activity of Verbascum caesareum. Chem. Nat. Compd..

[5] Pavel C.I., Mărghitaş L.A., Bobiş O., Dezmirean D.S., Şapcaliu A., Radoi I., Mădaş M.N. (2011). Biological Activities of Royal Jelly-Review. Sci. Pap. Anim. Sci. Biotechnol..

[6] Fratini F., Cilia G., Mancini S., Felicioli A. (2016). Royal Jelly: An ancient remedy with remarkable antibacterial properties. Microbiol. Res..

[7] Khazaei M., Ansarian A., Ghanbari E. (2018). New Findings on Biological Actions and Clinical Applications of Royal Jelly: A Review. J. Diet. Suppl..

[8] Ahmad S., Campos M.G., Fratini F., Altaye S.Z., Li J. (2020). New Insights into the Biological and Pharmaceutical Properties of Royal Jelly. Int. J. Mol. Sci..

[9] Buttstedt A., Moritz R.F.A., Erler S. (2013). More than royal food-Major royal jelly protein genes in sexuals and workers of the honeybee Apis mellifera. Front. Zool..

[10] Nagai T., Inoue R. (2004). Preparation and the functional properties of water extract and alkaline extract of royal jelly. Food Chem..

[11] Virgiliou C., Kanelis D., Pina A., Gika H., Tananaki C., Zotou A., Theodoridis G. (2019). A targeted approach for studying the effect of sugar bee feeding on the metabolic profile of Royal Jelly. J. Chromatogr..

[12] Sabatini A.G., Marcazzan G.L., Caboni M.F., Bogdanov S., Almeida-Muradian L.B. (2009). De Quality and standardisation of Royal Jelly. J. ApiProduct ApiMedical Sci..

[13] Balwierz R., Stojko J. (2020). Bee Products in Dermatology and Skin Care. Molecules.

[14] Vucevic D., Melliou E., Vasilijic S., Gasic S., Ivanovski P., Chinou I., Colic M. (2007). Fatty acids isolated from royal jelly modulate dendritic cell-mediated immune response in vitro. Int. Immunopharmacol..

[15] Collazo N., Carpena M., Nuñez-Estevez B., Otero P., Simal-Gandara J., Prieto M.A. (2021). Health Promoting Properties of Bee Royal Jelly: Food of the Queens. Nutrients.

[16] Laho M., Koh L., Mojžišov A., Majt J., Klaudiny J. (2018). 10-HDA, a Major Fatty Acid of Royal Jelly, Exhibits pH Dependent Growth-Inhibitory Activity Against Different Strains of Paenibacillus larvae. Molecules.

[17] Kawano Y., Makino K., Jinnin M., Sawamura S., Shimada S., Fukushima S., Ihn H. (2019). Royal jelly regulates the proliferation of human dermal microvascular endothelial cells through the down-regulation of a photoaging-related microRNA. Drug Discov. Ther..

[18] Bouamama S., Merzouk H., Latrech H., Charif N., Bouamama A. (2021). Royal jelly alleviates the detrimental effects of aging on immune functions by enhancing the in vitro cellular proliferation, cytokines, and nitric oxide release in aged human PBMCS. J. Food Biochem..

[19] Jiang C., Liu X., Li C., Qian H. (2018). Anti-senescence effect and molecular mechanism of the major royal jelly proteins on human embryonic lung fibroblast (HFL-I) cell line. J. Zhejiang Univ. SCIENCE B.

[20] Kunugi H., Ali A.M. (2019). Royal Jelly and Its Components Promote Healthy Aging and Longevity: From Animal Models to Humans. Int. J. Mol. Sci..

[21] Yang X.-Y., De-Sheng Y., Wei-Zhang, Wang J.-M., Li C.-Y., Hui-Ye, Lei K.-F., Chen X.-F., Shen N.-H., Jin L.-Q. (2010). 10-Hydroxy-2-decenoic acid from Royal jelly: A potential medicine for RA. J. Ethnopharmacol..

[22] Fujii A., Kobayashi S., Kuboyama N., Furukawa Y., Kaneko Y., Ishihama S., Yamamoto H., Toyoyuki T. (1990). Augmentation of Wound Healing by Royal Jelly (RJ) in Streptozotocin-Diabetic Rats. Jpn. J. Pharmacol..

[23] Dynek J.N., Chan S.M., Liu J., Zha J., Fairbrother W.J., Vucic D. (2008). Microphthalmia-Associated Transcription Factor Is a Critical Transcriptional Regulator of Melanoma Inhibitor of Apoptosis in Melanomas. Cancer Res..

[24] Chen C., Chen S.Y. (1995). Changes in protein components and storage stability of Royal Jelly under various conditions. Food Chem..

[25] Mendoza-Reséndez R., Gomez-Trevino A., Barriga-Castro E.D., Nunez N.O., Luna C. (2014). Synthesis of Antibacterial Silver-based Nanodisks and Dendritic Structures Mediated by Royal Jelly. RSC Adv..

[26] Complexes D.C., Saokham P., Muankaew C., Jansook P. (2018). Solubility of Cyclodextrins and Drug/Cyclodextrin Complexes. Molecules.

[27] Stella V., He Q. (2008). Cyclodextrins. Toxicol. Pathol..

[28] Eastburn S.D., Tao B.Y. (1994). Applications of Modified Cyclodextrins. Biotechnol. Adv..

[29] Szejtli J. (1988). Cyclodextrin Inclusion Complexes. Cyclodext. Technol.

[30] Nakhaei P., Margiana R., Bokov D.O., Abdelbasset W.K., Amin M., Kouhbanani J., Varma R.S., Maro F., Sarani M. (2021). Liposomes: Structure, Biomedical Applications, and Stability Parameters with Emphasis on Cholesterol. Front. Bioeng. Biotechnol..

[31] Lin E.Y., Chen Y.S., Li Y.S., Chen S.R., Lee C.H., Huang M.H., Chuang H.M., Harn H.J., Yang H.H., Lin S.Z. (2020). Liposome consolidated with cyclodextrin provides prolonged drug retention resulting in increased drug bioavailability in brain. Int. J. Mol. Sci..

[32] Yakavets I., Lassalle H., Scheglmann D., Wiehe A. (2018). Temoporfin-in-Cyclodextrin-in-Liposome—A New Approach for Anticancer Drug Delivery: The Optimization of Composition. Nanomaterials.

[33] Baldim I., Oliveira A.M., Souto E.B., Oliveira W.P. (2022). Cyclodextrins-in-Liposomes: A Promising Delivery System for Lippia sidoides and Syzygium aromaticum Essential Oils. Life.

[34] Cruz B.C., Flores M., Fernandez R.J.R., Vivas-mejia P.E., Barletta G.L. (2022). A Fresh Look at the Potential of Cyclodextrins for Improving the Delivery of siRNA Encapsulated in Liposome Nanocarriers. ACS Omega.

[35] Vafaei S.Y., Dinarvand R., Esmaeili M., Mahjub R., Toliyat T. (2015). Controlled-release drug delivery system based on fluocinolone acetonide—Cyclodextrin inclusion complex incorporated in multivesicular liposomes. Pharm. Dev. Technol..

[36] Papagiannaros A., Dimas K. (2005). Doxorubicin—PAMAM dendrimer complex attached to liposomes: Cytotoxic studies against human cancer cell lines. Int. J. Pharm..

[37] Demetzos C. (2015). Advanced Drug Delivery Nanosystems: Perspectives and Regulatory Issues.

[38] Spannhoff A., Kim Y.K., Raynal N.J., Gharibyan V., Su M., Zhou Y., Li J., Castellano S., Sbardella G., Issa J.J. (2011). Scientific report might facilitate caste switching in bees. EMBO Rep..

[39] Bucekova M., Sojka M., Valachova I., Ma S., Ranzato E., Szep Z., Majtan V., Klaudiny J., Majtan J. (2017). Bee-derived antibacterial peptide, defensin-1, promotes wound re-epithelialisation in vitro and in vivo. Sci. Rep..

[40] Antinelli J.-F., Zeggane S., Davico R., Rognone C., Faucon J., Lizzani L. (2003). Evaluation of (E)-10-hydroxydec-2-enoic acid as a freshness parameter for royal jelly. Food Chem..

[41] Li J., Feng M., Zhang L., Zhang Z., Pan Y. (2008). Proteomics analysis of major royal jelly protein changes under different storage conditions. J. Proteome Res.

[42] Katsnelson A. (2015). Cosmetics: Molecular beauty. Nature.

[43] Zhang W., Ge Y., Cheng Q., Zhang Q., Fang L., Zheng J. (2018). Decorin is a pivotal effector in the extracellular matrix and tumour microenvironment. Oncotarget.

[44] Danielson K.G., Baribault H., Holmes D.F., Graham H., Kadler K.E., Iozzo R.V. (1997). Targeted Disruption of Decorin Leads to Abnormal Collagen Fibril Morphology and Skin Fragility. J. Cell Biol..

[45] Park H.M., Hwang E., Lee G.K., Han S.-M., Cho Y., Kim S.Y. (2011). Royal Jelly Protects Against Ultraviolet B—Induced Photoaging in Human Skin Fibroblasts via Enhancing Collagen Production 1 1. J. Med. Food.

[46] Gupta A., Nitoiu D., Brennan-Crispi D., Addya S., Riobo N.A., Kelsell D.P., Mahoney M.G. (2015). Cell Cycle- and Cancer-Associated Gene Networks Activated by Dsg2: Evidence of Cystatin A Deregulation and a Potential Role in Cell-Cell Adhesion. PLoS ONE.

[47] Van Itallie C.M., Tietgens A.J., Anderson J.M. (2017). Visualizing the dynamic coupling of claudin strands to the actin cytoskeleton through ZO-1. Mol. Biol. Cell.

[48] Na J., Lee K., Na W., Shin J., Lee M., Yune T.Y., Lee H.K., Jung H., Kim W.S., Ju B. (2016). Histone H3K27 Demethylase JMJD3 in Cooperation with NF- k B Regulates Keratinocyte Wound Healing. J. Investig. Dermatol..

[49] Odorisio T. (2016). Epigenetic Control of Skin Re-Epithelialization: The NF-kB/JMJD3 Connection. J. Investig. Dermatol..

[50] Westergren-Thorsson G.U., Bagher M.A., Andersson-Sjöland A.N., Thiman L.E.N.A., Löfdahl C.L.Ö., Hallgren O.S., Bjermer L.E.I.F., Larsson-Callerfelt A.N.N.A.A. (2017). Original Article VEGF Synthesis is Induced by Prostacyclin and TGF-β in Distal Lung fi Broblasts from COPD Patients and Control Subjects: Implications for Pulmonary Vascular Remodelling. Respirology.

[51] Park M., Jang S., Chung J.H., Kwon O., Jo S.J. (2021). Inhibition of class I HDACs preserves hair follicle inductivity in postnatal dermal cells. Sci. Rep..

[52] Cabanel M., Pinheiro T., Cury M., Cheikh E.L., Carneiro K. (2019). The epigenome as a putative target for skin repair: The HDAC inhibitor Trichostatin A modulates myeloid progenitor plasticity and behavior and improves wound healing. J. Transl. Med..

[53] Detienne G., De Haes W., Ernst U.R., Schoofs L., Temmerman L. (2014). Royalactin extends lifespan of Caenorhabditis elegans through epidermal growth factor signaling. Exp. Gerontol..

[54] Honda Y., Fujita Y., Maruyama H., Araki Y., Ichihara K., Sato A. (2011). Lifespan-Extending Effects of Royal Jelly and Its Related Substances on the Nematode Caenorhabditis elegans. PloS ONE.

[55] Lee D., Lim J., Woo K., Kim K. (2018). Piperonylic acid stimulates keratinocyte growth and survival by activating epidermal growth factor receptor (EGFR). Sci. Rep..

[56] Li J., Cui L., Xu Y., Guan K. (2021). A Case of Anaphylaxis Caused by Major Royal Jelly Protein 3 of Royal Jelly and Its Cross-Reactivity with Honeycomb. J. Asthma Allergy.

[57] Rosmilah M., Shahnaz M., Patel G., Lock J., Rahman D., Masita A.N.A. (2008). Characterization of major allergens of royal jelly Apis mellifera. Trop. Biomed..

[58] Nakashima C., Otsuka A., Seidel J.A., Kabashima K. (2018). The effect of oral royal jelly administration on skin barrier function: A double-blind randomized placebo-controlled tria. Eur. J. Dermatol..

[59] Okumura N., Ito T., Degawa T., Moriyama M. (2021). Royal Jelly Protects against Epidermal Stress through Upregulation of the NQO1 Expression. Int. J. Mol. Sci..

[60] Gonzalez Gomez A., Syed S., Marshall K., Hosseinidoust Z. (2019). Liposomal Nanovesicles for Efficient Encapsulation of Staphylococcal Antibiotics. ACS Omega.

[61] Gardikis K., Hatziantoniou S., Signorelli M., Pusceddu M., Micha-Screttas M., Schiraldi A., Demetzos C., Fessas D. (2010). Thermodynamic and structural characterization of Liposomal-Locked in-Dendrimers as drug carriers. Colloids Surf. B Biointerfaces.

[62] Arnous A., Makris D.P., Kefalas P. (2002). Correlation of pigment and flavanol content with antioxidant properties in selected aged regional wines from Greece. J. Food Compos. Anal..

[63] Caparica-Santos C., Marcucci M.C. (2007). Quantitative determination of trans- 10-Hydroxy-2-Decenoic Acid (10-HDA) in Brazilian royal jelly and commercial products containing royal jelly. Apic. Res..

[64] Giampieri F., Alvarez-Suarez J.M., Mazzoni L., Forbes-Hernandez T.Y., Gasparrini M., Gonzàlez-Paramàs A.M., Santos-Buelga C., Quiles J.L., Bompadre S., Mezzetti B. (2014). Polyphenol-rich strawberry extract protects human dermal fibroblasts against hydrogen peroxide oxidative damage and improves mitochondrial functionality. Molecules.

[65] Letsiou S., Kalliampakou K., Gardikis K., Mantecon L., Infante C., Chatzikonstantinou M., Labrou N.E., Flemetakis E. (2017). Skin protective effects of Nannochloropsis gaditana extract on H_2_O_2_-stressed human dermal fibroblasts. Front. Mar. Sci..

[66] Ramakers C., Ruijter J.M., Lekanne Deprez R.H., Moorman A.F.M. (2003). Assumption-free analysis of quantitative real-time polymerase chain reaction (PCR) data. Neurosci. Lett..

[67] Lademann J., Jacobi U., Surber C., Weigmann H., Fluhr J.W. (2009). The tape stripping procedure—Evaluation of some critical parameters. Eur. J. Pharm. Biopharm..

